# Vertical scan imaging of Anterior Segment Optical Coherence Tomography for descemet anchoring caterpillar seta: A case report and review of literature

**DOI:** 10.1016/j.ajoc.2022.101671

**Published:** 2022-07-31

**Authors:** Mona Bhargava, Varsha G. Bhambhani, Raj Shekhar Paul

**Affiliations:** Department of Cornea, Aditya Birla Sankara Nethralaya, 147/1 E M Bypass Road, Kolkata, 700099, India

**Keywords:** Anterior segment optical coherence tomography (ASOCT) vertical scans, Caterpillar seta, Scheimpflug imaging, Seta migration

## Abstract

**Purpose:**

To report Anterior Segment Optical Coherence Tomography (ASOCT) characteristic and its usefulness in the case of Descemet's membrane (DM) anchoring caterpillar seta, highlighting the importance of vertical scans of ASOCT in detecting and delineating the deep seta and its anterior chamber (AC) penetration.

**Observation:**

A 30-year-old male patient presented with complaints of foreign body sensation and watering in the left eye after falling of an insect. Slit-lamp examination showed the presence of two caterpillar hairs, one embedded in the superficial conjunctiva and the second one embedded in the corneal stroma. Conjunctival hair was removed leading to the resolution of symptoms. ASOCT was done for deep corneal hair which showed DM breach and AC protrusion in volume vertical scans. ASOCT scan on follow-up visit showed migration of seta in AC. Deep seta being inert was not removed and the patient was closely followed.

**Conclusion:**

and IMPORTANCE: To the best of our knowledge, this is the first case reporting vertical scan imaging of caterpillar seta and showing AC migration of seta on ASOCT.

## 1 Introduction

Ophthalmia nodosa is defined as an inflammatory reaction in the eye to hairs of certain insects or vegetable material and derives its name from the nodular conjunctival reaction that they produce.^1^ The first description was published by Schon in 1861. Later on, many cases have been recorded in literature from time to time during the last 120 years.^1^ Diagnosis and management of hairs embedded in the cornea have been mostly clinical based on slit-lamp examination. It is subjective and variable and depends significantly on the experience of the examiner.^2^ Technology has revolutionized the field of medicine with the availability of newer investigative modalities like Anterior segment optical coherence tomography (ASOCT), Ultrasound biomicroscopy (UBM), In-Vivo confocal microscopy (IVCM) and Scheimpflug imaging. Thus the approach to diagnosis and management changed with the above-mentioned modalities providing a reliable and objective method for documentation, assessment and monitoring of the pathology as well as a powerful tool for patient learning and understanding. There is a scarcity of literature citing the use of such modalities for detecting, diagnosing and monitoring caterpillar hair in the cornea. We report ASOCT characteristics and their usefulness in a case of Descemet's membrane (DM) anchoring caterpillar seta.

## 2 Case description

**Fig. 1:**
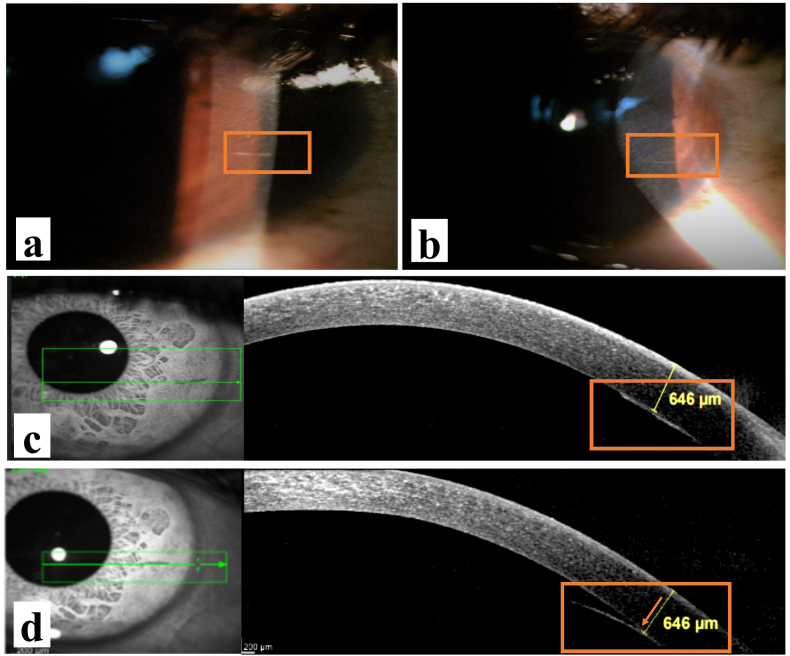
Slit-lamp photograph and horizontal ASOCT scan images (a),(b)-caterpillar hair embedded in the cornea with surrounding clear cornea. Noteworthy is the absence of any DM striae, corneal edema or infiltrate; (c)-ASOCT horizontal scan on presentation showing linear hyper-reflective seta anchoring Descemet's with the compact overlying cornea of −646 μm thickness; (d)- ASOCT horizontal scan at follow-up visit showing 2/3 floating nasal (distal) end of the hair in AC with 1/3 temporal (proximal) end embedded in the cornea. Note that the overlying cornea maintains its compactness with a thickness of 646 μm. Orange arrow shows the site of DM breach. . (For interpretation of the references to colour in this figure legend, the reader is referred to the Web version of this article.)

**Fig. 2:**
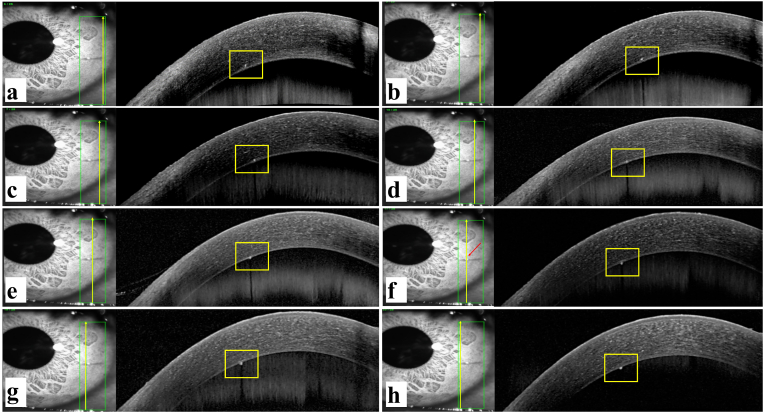
ASOCT vertical scan depicting the migration of seta from cornea to anterior chamber (a),(b),(c)- ASOCT vertical scan images showing hyper-reflective spot corresponding to seta location in the cornea; (d),(e)- ASOCT vertical scan images showing hyper-reflective spot at DM level corresponding to seta traversing DM; (f)- ASOCT vertical scan image showing hyper-reflective spot in AC for the first time corresponding to site where seta after breaching DM now enters AC. The red arrow is at nasal 1/3rd and temporal 2/3rd junction; (g),(h)- ASOCT vertical scan images showing hyper-reflective spot corresponding to seta location in AC. The central vertical yellow line in Fig. 2a to h shows the site of intersection of vertical scan with a length of setae for each scan. . (For interpretation of the references to colour in this figure legend, the reader is referred to the Web version of this article.)

A 30-year-old male patient presented with complaints of mild foreign body sensation and watering in the left eye for the past 15 days. He had a history of insect fall in the left eye (OS) and subsequent multiple attempts of caterpillar hair removal in the past 15 days. His visual acuity was 6/6 and N6 in both eyes (OU). His intraocular pressures for OU were within the normal range in all visits. Slit-lamp examination of the right eye was unremarkable, left eye examination showed two caterpillar hairs, one in superficial bulbar conjunctiva in the temporal inter-palpebral region, and another in the peripheral cornea at 3 o'clock (Fig. 1a and b). Corneal hair was embedded in the deep stromal layer and had no edema or infiltration around it suggestive of any associated keratitis. There was no cell or flare reaction in AC in OS. ASOCT of deep corneal hair done using the Spectralis Anterior Segment Module (Heidelberg Engineering GmbH, Heidelberg, Germany) showed one hyper-reflective linear echo corresponding to hair shaft anchoring Descemet's membrane (DM) in horizontal scans (Fig. 1c). As the hair followed the arc and curvature of DM, it was difficult to comment on any AC protrusion with DM breach. So, volume vertical scans were done through the entire length of hair in which hair appeared as hyper-reflective spot echo (Fig. 2). It showed progressive posterior migration of hyper-reflective spot from posterior stroma just above DM; at the level of DM and below the level of DM. Fig. 2a,b, and 2c show a hyper-reflective spot above the DM indicating that in temporal (proximal) length, seta was in posterior stroma just above the DM. Fig. 2d and e shows a hyper-reflective spot at the level of DM depicting that in that small length seta traverses DM. Fig. 2f shows a hyper-reflective spot below the DM for the first time; the site where setae now after breaching the DM enters AC; which is at the junction of nasal (distal) 1/3rd and temporal (proximal) 2/3rd of the setae length. Fig. 2g and h shows hyper-reflective spot lying below the DM suggestive that setae in nasal (distal) 1/3rd is in AC following the arc of DM. So, a diagnosis of deep corneal caterpillar hair breaching DM with 1/3 of the nasal (distal) end in AC was made (Fig. 2). Hair in the conjunctiva was removed on a slit-lamp under topical anesthesia with sterile precautions and the patient was started on topical dexamethasone (0.1%) in a tapering dose along with topical moxifloxacin (0.5%) and topical carboxy-methylcellulose (0.5%). The patient was kept under observation for any signs and symptoms of inflammation. Two months later the patient was asymptomatic and the eye was quiet but the hair appeared to have a floating end in AC. Follow-up ASOCT showed 2/3 floating nasal (distal) end of the hair in AC with 1/3 temporal (proximal) end still anchored to posterior stroma (Fig. 1d). Any invasive surgery for deep corneal hair was not opted due to quiet eye and the anticipated difficulty in removal of fragile hair. The patient was kept under observation for any further migration of corneal hair. UBM and Ultrasound sonography (USG) of OS was done to locate any posterior hair and were unremarkable. Fundus examination of both eyes was within normal limits.

## 3 Discussion

Caterpillars are the larval form of a member of the insect order Lepidoptera, which includes butterflies and moths.^2^ The various types of caterpillars incriminated are representatives of Lasiocampa/Bombyx, Enethocampa and Liparida. Depending on the life cycle of the caterpillar, keratoconjunctivitis has a seasonal incidence in autumn. Contact with caterpillar setae can occur through direct contact with caterpillars, contact with the larval cocoon into which setae may be shed and get interwoven, contact with adult Lepidoptera that may carry larval setae on their bodies, direct reaction to adult setae and borne wind setae.^3^ Penetration of the cornea results in localized or nummular keratitis, depending on the number of hairs involved. Thereafter, there is a quiet interval lasting for a few days, the period during which hair migrates through the cornea.^4^ Scanning electron microscopy observation of removed setae revealed that each seta had spines directed towards the tip, allowing only tip-ward penetration. The deep penetration of setae is suggested to be related to the movement of the globe with versions, respiration, and pulse together with constant iris movement, which propels the spines forward; the setae themselves do not have any propulsive power.^3^ The quiescent stage may be followed by a phase of inflammation when the hair is free or protruding in AC or is irritating the anterior uvea. Sometimes, this reaction may be sufficiently severe to produce a hypopyon and nodules on the iris or flat yellow and oval nodules in the conjunctiva.^4^

Pathology caused by setae is due to (a) mechanical locomotive damage of setae and (b) a toxic irritating mechanism of the urticant hairs.^3^^,^^5^ Urticating hair is composed of sharp setae associated with poison glands under the setal base. When a seta is ruptured by trauma, the poison will be released.^6^ The urticating protein of the pine processionary caterpillar has been identified as thaumetopoein, and a similar protein may exist in other caterpillars.^5^^,^^6^ (c) Allergic hypersensitivity mechanism mediated by immunoglobulin E (IgE) as reported by a few authors. Several proteins, most with molecular weights of 13, 15 and 17 kDa are responsible for the allergic reaction.^5^

The ocular symptomatology can also be separated into immediate IgE-dependent and delayed hypersensitivity processes. The IgE-mediated process generally produces red-eye, punctate keratitis, and itchiness on the ocular surface. The delayed corneal reactions are produced in response to the embedded caterpillar hairs. The reaction appears as stromal infiltrate around the hairs days after contact. These infiltrates are similar to subepithelial infiltrates characteristic of viral keratoconjunctivitis. They surround the hair progressively causing the patient to have different symptoms depending on the area of corneal involvement. Central corneal involvement causes worsening of visual symptoms and photophobia. However, with only peripheral involvement, the patient does not usually have any visual complaints.^5^ Histopathology of the lesions shows a typical granulomatous reaction to a chemical irritant. The setae are surrounded by lymphoid cells, macrophages, and epithelioid cells encircled by a thick fibrous capsule.^3^

In clinical practice, imaging of corneal lesions has traditionally been carried out with slit-lamp biomicroscopy. However, its use for objective quantitative assessment of anterior segment structures is limited.^2^ ASOCT provides cross-sectional anatomical images of the cornea and anterior segment. Caterpillar setae appear as a hyperreflective structure on ASOCT. Horizontally-oriented setae will appear as a linear hyper-reflective structure on horizontal scans (Fig. 1c and d) and dot hyper-reflective structure on vertical scans (Fig. 2). This hyper-reflective echo can outline exact depth, location, size, orientation, and any obliquity as well as any DM breach. In our patient horizontal scans at the first visit showed one deep stromal DM anchoring seta which posed difficulty in visualizing any breach in DM integrity and tracing AC protrusion (Fig. 1c). So vertical scans were also done which showed a hyperreflective spot echo corresponding to seta. Serial scans showed gradual posterior progression of spot echo in the corneal stroma (Fig. 2a–c) followed by the echo breaching DM (Fig. 2d and e) and then in AC (Fig. 2f–h). Not only AC penetration of hair was evident, but the exact site of DM breach could also be marked, allowing quantification of the proportion of hair in AC versus in stroma. ASOCT is also a good tool for close monitoring of migration of setae. ASOCT on follow-up visits of the same patient showed progressive AC migration of the same seta with a major length of hair in AC and a small portion anchored to the cornea.

When surrounded by edema, infiltrate or opacity; fine setae can be missed clinically but are picked up on the investigative modalities like ASOCT, IVCM, Scheimpflug imaging and UBM. Setae appear as hyperreflective echoes on all imaging modalities. Timucin OB et al., have described the use of scheimpflug imaging as a tool for localization and monitoring of keratitis caused by superficial caterpillar seta.^2^ We could not find any literature citing the use of Scheimpflug for monitoring caterpillar migration on PubMed search.

In vivo confocal microscopy (IVCM) offers the opportunity to extract an en-face microstructure image at the cellular level in real-time.^7^ Jullienne R et al., have described IVCM characteristic of Pine Processionary Caterpillar Hair as a linear highly hyper-reflective foreign body with serrated edges corresponding to small spikes visible at the edges and oriented toward the tip.^8^ Bartolomé F et al. were able to pinpoint hair using IVCM, which was not delineated by ASOCT in one of their cases.^9^ In absence of surrounding corneal edema or infiltrate ASOCT would usually pick a superficial lying corneal foreign body. We suggest that a combination of both horizontal and vertical scans is a better option for delineation of obliquely oriented hair seen in their series. In comparison to ASOCT, IVCM provides a higher lateral resolution of approximately 1.0 μm but with a significantly smaller field of view. Although it is possible to image all corneal layers, the focus can only be set to one plane of interest at a time and different corneal layers have to be imaged separately. Another disadvantage is that it is a contact technique that induces discomfort in patients exacerbating symptoms and carries a small risk of corneal epithelial defects.^7^^,^^9^ Also, it requires specific training and the image interpretation is subjective.^7^^,^^9^

Few authors have reported the use of UBM to locate caterpillar hair in the anterior segment as well as pars plana.^10^ But its use is limited as it is a contact procedure requiring water-bath coupling media and a very experienced examiner.^10^ ASOCT is a non-contact, efficient and user-friendly clinical tool.^7^

Cadera et al. have classified the ocular pathology of caterpillar setae into five types. Type 1 (Acute toxic reactions), Type 2 (Chronic mechanical keratoconjunctivitis), Type 3 (granulomatous nodules under conjunctiva), Type 4 (Iritis/iris nodules), Type 5 (Vitreoretinal involvement). Based on this classification, the suggested treatment modalities include the following: Type 1/2 Irrigation followed by meticulous removal of setae. Topical antibiotics and steroids. Type 3 Surgical excision of nodules. Type 4 Topical steroids ± iridectomy for nodules or operative removal of setae. Type 5 Local ± systemic steroids. Resistant cases-vitrectomy with removal of setae.^3^ The largest series by Sengupta et al. reported mild conjunctival involvement as the most frequent (87%), followed by corneal involvement (27%) and Vitreo-retinal manifestations in only 2.4% of cases.^11^ The majority of the cases fall between type1-4.^11^^,^^12^ Intraocular migration of setae is a known complication and can lead to severe anterior or posterior segment reaction. However, most of the cases respond well to steroids and cycloplegic treatment.^11^^,^^13^ Frank endophthalmitis is rare but reported.^11^ Horng et al. have described an excellent clinical course without complication in their patient where five setae near the endothelium of the cornea, anterior chamber, and anterior iris surface were left in place because they were out of the visual axis and without any apparent toxic sign.^6^ Ibarra et al. also managed setae conservatively and concluded that intraretinal and corneal setae can be embedded with minimal inflammation and can be tolerated without the need for surgical intervention.^12^ Nicholas et al. also adopted a conservative approach for deep corneal setae, given the patient had no visual loss and the risks of corneal surgery to remove a single seta outweighed the benefits.^14^ Sridhar et al. in their series of ten patients reported that none of the patients with setae protruding into the anterior chamber had such an intense reaction as to warrant surgical intervention.^3^ Portero et al. do not recommend the removal of intracorneal hairs due to the risk of mechanical trauma to the cornea. Also, they did not find any intraocular penetration in the past 30 years and all their patients healed successfully with or without medications.^5^ As our patient had absolutely no signs of inflammation and considering the surgical difficulty of removing single DM anchoring setae in a quiet eye with the possibility of iatrogenic endothelial insult and slippage of setae intraoperative, a decision to conservative management with close follow-up was undertaken after the patient was acquainted about possible late migration and complication. Despite it, the authors firmly believe in the removal of all amenable superficial and deep setae and granulomas. Also, surgical removal in form of AC retrieval or pars-plana vitrectomy would be the only option in recalcitrant or vision-threatening cases.^5^ In cases where a conservative approach is chosen, close follow-up is mandatory where ASOCT comes along as a handy tool.

ASOCT can thus be used to assess the depth of the hairs inside the cornea, which was described as the main risk factor for intraocular penetration. It helps in the easy management of any hair which is potentially accessible to surgical removal.^11^ Thus ASOCT helps in delineation, depth assessment, identification of any breach in DM integrity, anterior chamber protrusion, monitoring migration and thereby follow-up for any residual setae sequelae.

## 4 Conclusion

Slit-lamp examination techniques are of utmost importance for delineating anterior segment foreign bodies, but ASOCT acts as an important adjunct for localization and migration of fine, hair-like, linear, refractive foreign bodies such as caterpillar seta, whenever in doubt. We document ASOCT characteristics and posterior migration of caterpillar hair via horizontal and vertical scans highlighting their usefulness and importance which has not been documented before. Newer modalities like ASOCT, Scheimpflug imaging and IVCM provide an objective and accurate assessment of setae and its associated complications and form a puissant tool for patient education.

## 5 Patient consent

Consent to publish this case report has been obtained from the patient in writing - the authors certify that they have obtained all appropriate patient consent forms. In the form, the patient has given his consent for his images and other clinical information to be reported in the journal. The patient understands that his name and initials will not be published and due efforts will be made to conceal the identity.

## Funding

No funding or grant support

## Authorship

All authors attest that they meet the current ICMJE criteria for authorship.

## Source(s) of support

Nil.

## Financial support

Nil.

## Prior presentation

None.

## Declaration of competing interest

The following authors have no financial disclosures: Mona Bhargava, Varsha Bhambhani, Raj Shekhar Paul.
